# Catalpol reduced LPS induced BV2 immunoreactivity through NF-κB/NLRP3 pathways: an *in Vitro* and in silico study

**DOI:** 10.3389/fphar.2024.1415445

**Published:** 2024-06-27

**Authors:** Yong She, Chong-yu Shao, Yuan-feng Liu, Ying Huang, Jiehong Yang, Hai-tong Wan

**Affiliations:** ^1^ School of Life Sciences, Zhejiang Chinese Medical University, Hangzhou, Zhejiang, China; ^2^ School of Basic Medical Sciences, Zhejiang Chinese Medical University, Hangzhou, Zhejiang, China

**Keywords:** neuroinflammation, microglia, molecular docking analysis, molecular dynamics simulation, *in vitro*

## Abstract

**Background:** Ischemic Stroke (IS) stands as one of the primary cerebrovascular diseases profoundly linked with inflammation. In the context of neuroinflammation, an excessive activation of microglia has been observed. Consequently, regulating microglial activation emerges as a vital target for neuroinflammation treatment. Catalpol (CAT), a natural compound known for its anti-inflammatory properties, holds promise in this regard. However, its potential to modulate neuroinflammatory responses in the brain, especially on microglial cells, requires comprehensive exploration.

**Methods:** In our study, we investigated into the potential anti-inflammatory effects of catalpol using lipopolysaccharide (LPS)-stimulated BV2 microglial cells as an experimental model. The production of nitric oxide (NO) by LPS-activated BV2 cells was quantified using the Griess reaction. Immunofluorescence was employed to measure glial cell activation markers. RT-qPCR was utilized to assess mRNA levels of various inflammatory markers. Western blot analysis examined protein expression in LPS-activated BV2 cells. NF-κB nuclear localization was detected by immunofluorescent staining. Additionally, molecular docking and molecular dynamics simulations (MDs) were conducted to explore the binding affinity of catalpol with key targets.

**Results:** Catalpol effectively suppressed the production of nitric oxide (NO) induced by LPS and reduced the expression of microglial cell activation markers, including Iba-1. Furthermore, we observed that catalpol downregulated the mRNA expression of proinflammatory cytokines such as IL-6, TNF-α, and IL-1β, as well as key molecules involved in the NLRP3 inflammasome and NF-κB pathway, including NLRP3, NF-κB, caspase-1, and ASC. Our mechanistic investigations shed light on how catalpol operates against neuroinflammation. It was evident that catalpol significantly inhibited the phosphorylation of NF-κB and NLRP3 inflammasome activation, both of which serve as upstream regulators of the inflammatory cascade. Molecular docking and MDs showed strong binding interactions between catalpol and key targets such as NF-κB, NLRP3, and IL-1β.

**Conclusion:** Our findings support the idea that catalpol holds the potential to alleviate neuroinflammation, and it is achieved by inhibiting the activation of NLRP3 inflammasome and NF-κB, ultimately leading to the downregulation of pro-inflammatory cytokines. Catalpol emerges as a promising candidate for the treatment of neuroinflammatory conditions.

## 1 Introduction

Stroke is a severe cardiovascular disease posing significant threats to human health, characterized by high incidence, disability, and mortality rates. Stroke is the second leading cause of disability and death globally, with low- and middle-income countries bearing the greatest burden. In 2016, there were 13.7 million new cases of stroke worldwide, with approximately 87% being ischemic strokes (Saini et al., 2021). Overall, stroke accounted for 11.6% of total deaths globally, reinforcing its status as a major public health concern (Feigin et al., 2021). An increasing body of evidence suggests that neuroinflammation is associated with the primary pathogenesis of ischemic stroke. (Maida et al., 2020). During the entire inflammatory process, various immune cells, including microglia and macrophages, become activated (Iadecola and Anrather, 2011). Among these innate immune cells, microglia take the central stage in the realm of neuroinflammation (DiSabato et al., 2016). After injury or infection, microglia shift towards a phenotype that promotes inflammation, such as exposure to bacterial-derived products like LPS. Thus, the cells release pro-inflammatory cytokines like TNF-α, interleukin (IL)-1β, and IL-6, along with inducible NO (iNOS) that contributes to nitric oxide creation (Orihuela et al., 2016). Therefore, controlling microglial activation and the inhibition of pro-inflammatory mediators and cytokines are viewed as promising treatment strategies in the onset and progression of ischemic stroke.

*Rehmannia glutinosa,* a commonly used herb in traditional Chinese medicine, has been widely employed to treat diabetes (Bian et al., 2023), neurological disorders (Pan et al., 2022), and inflammation (Zhang et al., 2023). Catalpol (CAT), an iridoid glycoside compound isolated from *Rehmannia glutinosa*, is a water-soluble component known for its anti-inflammatory and hepatoprotective properties. (Zhang J. et al., 2019). Recent studies have demonstrated the anti-inflammatory properties of catalpol in various conditions such as diabetes (Shu et al., 2021), nephropathy (Zaaba et al., 2023), and encephalopathy. In the context of ischemic stroke, *in vitro* experiments have utilized different cell types to study the effects of catalpol. These cell types include SY5Y cells, primary neurons, BMEC cells, and BV2 microglia, highlighting its potential therapeutic application across a range of neural and non-neural cell models. Nonetheless, the efficacy of catalpol in effectively mitigating the activation of the LPS-induced microglial remains to be established, and the underlying molecular mechanisms for anti-neuroinflammation remain unexplored.

In this investigation, we used BV2 cells treated with LPS, a mouse microglial cell line, as an experimental model to explore the anti-inflammatory characteristics of catalpol and to clarify the signaling pathways involved in its effects. To identify key catalpol targets, we used molecular docking to predict binding affinity with specific proteins. This was followed by validation through molecular dynamics (MD) simulation techniques and further *in vitro* experiments. Collectively, our findings strongly indicate that catalpol has a significant impact on neuroinflammatory responses induced by LPS in BV2 microglial cells.

## 2 Materials and methods

### 2.1 Cell lines and cell culture

Mouse microglial cells (BV2, Accession Number: CVCL_0182) were obtained from the Cell Bank of Type Culture Collection of the Chinese Academy of Sciences (Shanghai, China) and were cultured in a 37°C, 5% CO_2_ environment using high-glucose DMEM (Gibco, ThermoFisher Scientific, US), supplemented with 10% fetal bovine serum (FBS, BI, Israel), and 1% penicillin/streptomycin. The subsequent experiments were conducted using BV2 cells at passages 3–5. Upon reaching 80% confluence, the cells were split using 0.25% trypsin and sub-cultured for subsequent experiments. Cells were pretreated with catalpol (250 and 500 μM) for 24 h, followed by treatment with or without LPS (500 ng/mL) for 6 h. The catalpol (product number: AB1238-100 mg; CAS number: 2415-24-9; HPLC ≥98%) was purchased from Chengdu Alfa Biotechnology Co., Ltd.

The BV2 cells were divided into four groups for subsequent experimental studies: the blank control group (C group), the model group (M group), the catalpol low-dose group (CAT-L group), and the catalpol high-dose group (CAT-H group).

### 2.2 CCK-8 cell viability assay

A CCK-8 assay followed the manufacturer’s instructions with a commercial kit from Biosharp, Guangzhou, China. (Zhao et al., 2023). In summary, BV2 cells were plated in 96-well plates during the logarithmic growth phase (1 × 10^5^ cells/well) and exposed to varying concentrations of catalpol (50–2000 μM) for 24 h. Subsequently, The cells were exposed to 10 μL/well of the CCK-8 solution and then placed in a 5% CO_2_ incubator at 37°C for 1 hour, after which the absorbance was measured at 450 nm. For each concentration of catalpol, six technical replicates were used, and the entire experiment was repeated three times.

### 2.3 NO assay

50 μL/well of supernatants were collected, and the levels of total nitric oxide (NO) were determined using the Griess assay kit (Beyotime, Shanghai, China) by the manufacturer’s instructions, as previously reported. The sodium nitrite standard was diluted in complete culture medium to concentrations of 0, 1, 2, 5, 10, 20, 40, 60, and 100 μM, and a standard curve was constructed. To calculate nitrite concentrations, sodium nitrite was used to create a standard curve of different dilutions. The reaction mixtures were measured for their absorbance at 540 nm using a microplate reader.

### 2.4 Real-time quantitative PCR (RT-qPCR)

Harvested BV2 cells were subjected to total RNA extraction using the FastPure Cell/Tissue Total RNA Isolation Kit V2 (Vazyme; Nanjing, China). The RNA was reverse transcribed (Biosharp, Guangzhou, China), and quantitative assessment (ABclonal, Wuhan, China) was performed as previously described (Yu et al., 2022). The amplification reactions were performed according to the ABclonal qPCR reagent kit instructions. The cycling conditions were 95°C for 3 min, followed by 40 cycles of 95°C for 5 s and 60°C for 30 s. GAPDH was used as the housekeeping gene, and the relative expression levels of the target genes were calculated using the 2^−ΔΔCt^ method with the control group as the reference. The primer sequences can be found in Supplementary Table S1.

### 2.5 Western blotting

Western blotting was performed as previously described (Zhao et al., 2023). Sixteen micrograms of protein were separated using sodium dodecyl sulfate-polyacrylamide gel electrophoresis (SDS-PAGE) and then transferred onto polyvinylidene fluoride (PVDF) membranes. After transfer, PVDF membranes were blocked with 5% BSA for 1.5 h and incubated overnight at 4°C with primary antibodies. The next day, the membranes were incubated with a secondary antibody for 1.5 h. The information about the antibodies is shown in Supplementary Table S2.

### 2.6 Immunofluorescence staining

The experimental procedure was performed as previously described (Zhao et al., 2023). The cells were fixed in 4% paraformaldehyde at room temperature for 30 min and then washed thrice with PBS. After permeabilization with 0.2% Triton X-100/PBS for 15 min, the cells were washed with PBS, blocked in PBS with 5% BSA at room temperature for 1 h, and then incubated overnight at 4°C with primary antibodies. The next day, after being washed with PBS, the cells were incubated with ABflo^®^ 594 goat anti-rabbit (1:200) secondary antibody for 1 h in the dark. Then, the cells were washed with PBS and treated in the dark with an antifade mounting medium containing DAPI for 10 min at 37°C. Finally, the relative positive expression rates of the sections were determined using ImageJ, and information about the antibodies is shown in Supplementary Table S2.

### 2.7 Annexin V-FITC (apoptosis) assay

Apoptosis was assessed by flow cytometry using the Annexin V-FITC Apoptosis Detection kit (Beyotime, Shanghai, China) and performed according to the manufacturer’s instructions. The cells were detached using trypsin and then pelleted by centrifugation at 1,000 rpm for 5 min. The obtained cells were washed with ice-cold PBS (1X) and resuspended in 400 μL binding solution. Next, the cells were stained with both Annexin V-FITC (5 μL) and propidium iodide (10 μL) in the dark for 20 min at room temperature, and their apoptosis rate was determined within 0.5 h using a flow cytometer (Beckman, US). Data were analyzed using FlowJo software.

### 2.8 Database websites and software

The databases and software used were as follows: PubChem database (https://pubchem.ncbi.nlm.nih.gov/), GeneCards (http://www.genecards.org), UniProt (https://www.uniprot.org), Acpype Server (https://www.bio2byte.be/acpype/), Autodock vina_1.2.3 software, Discovery Studio 2021 software, Gromacs 2019.6, and Gmx_MMPBSA software.

### 2.9 Molecular docking

The protein structure files of IL-1β, NLRP3, and NF-κB were obtained from AlphaFold. The three-dimensional (3D) structure of Catalpol was retrieved from the PubChem database. Following the method of Liu et al. (2024), We used AutoDockTools to prepare the three-dimensional structures of key components and the crystal structures of core proteins. Subsequently, molecular docking of the ligands and receptors was performed using AutoDock Vina. Finally, visual analysis was conducted using PyMOL and Discovery Studio.

### 2.10 Molecular dynamics simulation

After docking, the MD simulation was performed using Gromacs 2022.2 software, the AMBERff14SB force field, and the TI3P model to check the stability of the combined pose with the lowest binding energy. The complexes were placed in a dodecahedral box with a minimum distance of 1.2 nm between the protein atoms and the box edges. Energy minimization was performed using the steepest descent method until the energy converged to 1,000 kJ mol^-1^ nm^-1^. Finally, the simulation trajectories were analyzed using various scripts integrated within GROMACS.

### 2.11 Binding energy calculation through MM-PBSA

The binding free energy of protein-ligand complexes was calculated using the g_mmpbsa tool, which calculated the binding free energy of complex structures using the Molecular Mechanics Poisson–Boltzmann surface area (MM-PBSA) method.

### 2.12 Statistical analysis

Results are expressed as mean ± SD. Statistical analysis of experimental data was performed using one-way analysis of variance (one-way ANOVA) on the results of at least three independent biological replicates. Multiple comparisons were subsequently performed using the Tukey test. All calculations were performed using GraphPad Prism 10 (GraphPad Software, San Diego, CA). *p* < 0.05 was considered statistically significant.

## 3 Results

### 3.1 Effects of catalpol on the viability of BV2 cells

**FIGURE 1:**
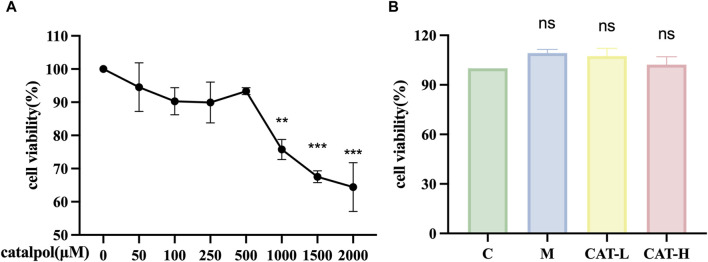
The effects of catalpol on proliferation in BV2 cells. CCK-8 assay was used to detect the viability of BV2 cells. **(A)** BV2 cells were treated with catalpol (0, 50, 100, 250, 500, 1,000, 1,500 or 2,000 μM) for 24 h **(B)**. BV2 cells were pretreated with 250 μM catalpol (CAT-L) or 500 μM catalpol (CAT-H) for 24 h, and then incubated with or without LPS (500 ng/mL) for 6 h.

To assess the impact of catalpol on BV2 cell viability, a CCK-8 assay was performed after treating with varying concentrations of catalpol (50–2000 μM) for 24 h. The results (Figure 1A) demonstrated that catalpol concentrations below 1 mM did not cause observable cytotoxic effects. Cytotoxic effects were observed above 1.5 mM. Consequently, concentrations of catalpol within the range of 250–500 μM were utilized in subsequent experiments. The BV2 cells underwent pretreatment with varying concentrations of catalpol for 24 h, followed by a 6-hour incubation period with LPS (500 ng/mL); the model group was not pretreated with catalpol, and the control group received no treatment. Catalpol showed no significant toxic effects on cell viability at different concentrations (250 and 500 μM) (Figure 1B).

### 3.2 Effects of catalpol on the production of NO inflammatory cytokines in LPS-induced BV2 cells

**FIGURE 2:**
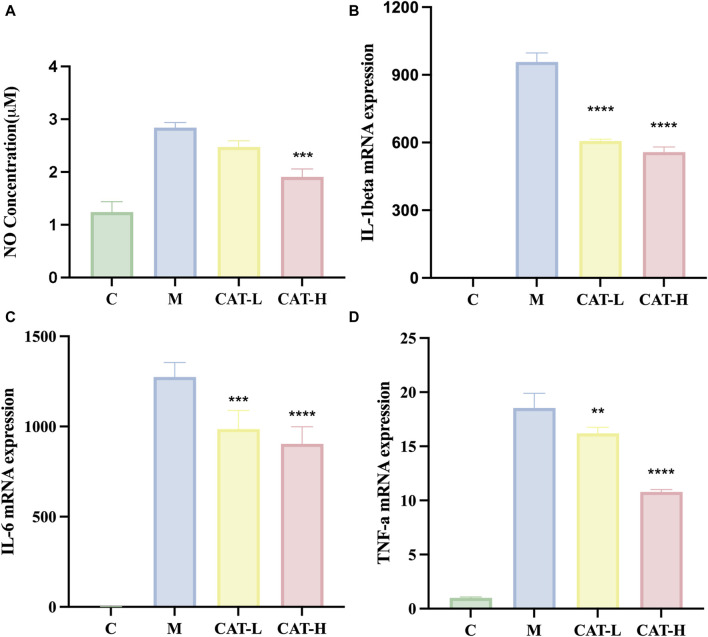
**(A)** NO concentration in the supernatant was measured using the Griess reaction. Gene expression levels of IL-1β, IL-6, and TNF-αwere assessed using RT-qPCR. **(B–D)**. All data are presented as the mean ± SD (*n* = 3). **p < 0.01, ***p < 0.001 ****p < 0.0001.

Nitric oxide (NO) serves as a crucial indicator of inflammation, playing a pivotal role in diverse manifestations of inflammation and carcinogenesis. (Choi et al., 2008). Hence, measuring NO production can offer insights into the impact of catalpol on the inflammatory process. The experimental procedure involved pretreating BV2 cells with different concentrations of catalpol for 24 h, followed by a 6-hour incubation period with LPS. The results, shown in Figure 2A, revealed a noteworthy increase in NO levels following LPS treatment in comparison to the control group. Nonetheless, the treatment of catalpol considerably reduced the production of NO induced by LPS.

### 3.3 Effects of catalpol on the level of proinflammatory cytokines in LPS-induced BV2 cells

We performed RT-qPCR experiments to explore the plausible regulatory impacts of catalpol on proinflammatory responses. Total RNA was isolated, and the levels of proinflammatory cytokines IL-1β, IL-6, and TNF-α were quantified. The results (Figure 2) demonstrated a noteworthy elevation in the concentrations of pro-inflammatory cytokines following LPS stimulation, as evidenced by the increased production of IL-1β, IL-6, and TNF-α. Importantly, catalpol treatment mitigated the synthesis of pro-inflammatory cytokines triggered by LPS. These findings suggest that catalpol demonstrated anti-inflammatory properties in BV2 cells stimulated by LPS.

**FIGURE 3:**
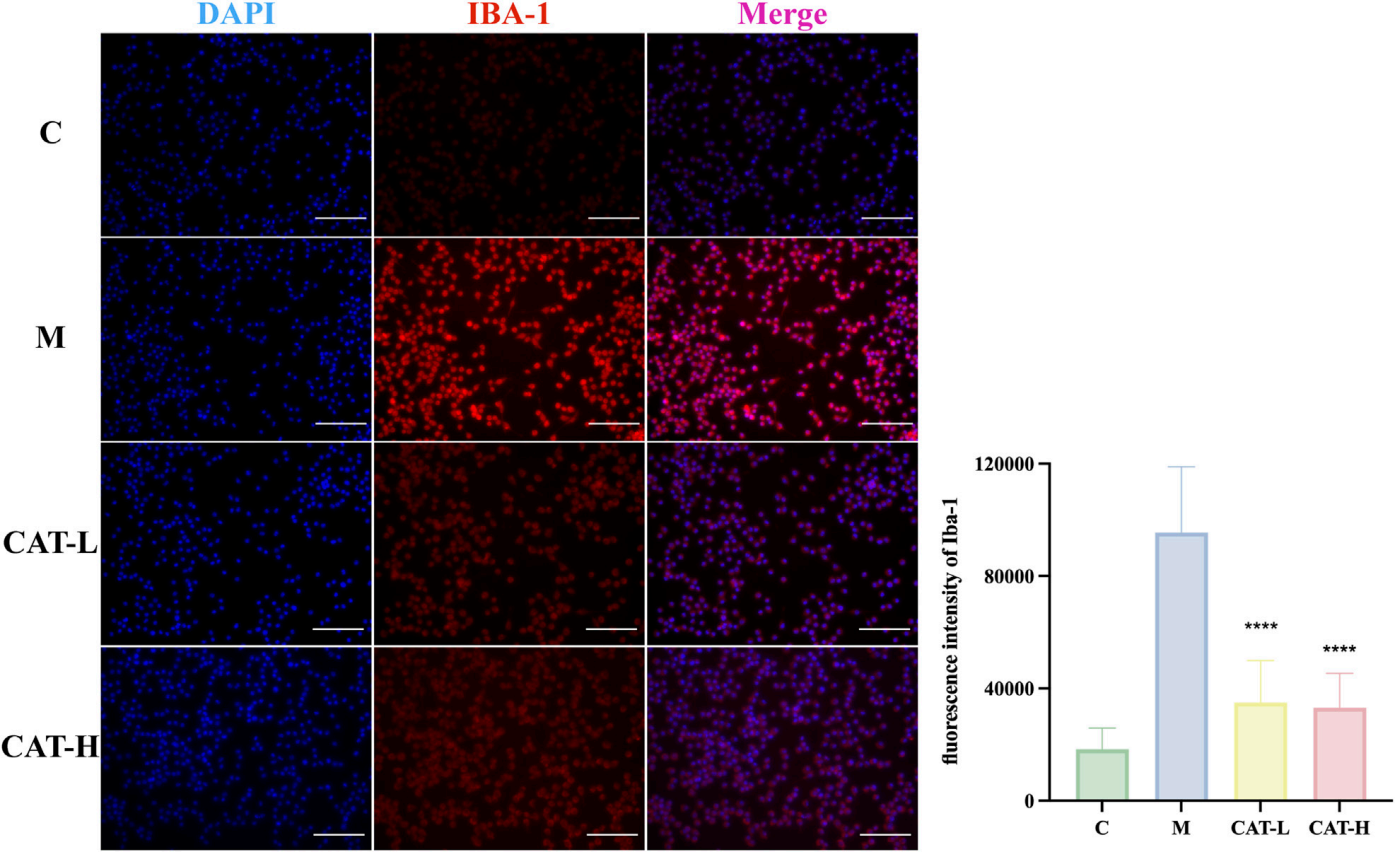
Cell morphology and IBA-1 protein expression were observed with immunofluorescence staining. Representative images of IF staining at × 20 magnification. Representative photomicrographs showed BV-2 cells labeled with an Iba-1 antibody (in red), with cell nuclei counterstained using DAPI (in blue). Scale bar: 500 μM. All data are presented as the mean ± SD (*n* = 3). ****p < 0.0001.

As activated microglia are recognized for releasing proinflammatory mediators within the brain parenchyma, we investigated the potential influence of catalpol on microglial activation. Specifically, we focused on Iba-1, a calcium-binding protein that exhibits specific expression in microglia and is upregulated upon their activation. Our findings revealed that the LPS-treated group exhibited pronounced microglial hypertrophy and a significant increase in microglial activation compared to the untreated control group. Following LPS treatment, microglia adopted a pro-inflammatory amoeboid morphology, featuring thick and short cell bodies. In contrast, after treatment with catalpol, the BV2 cells morphological changes induced by LPS were attenuated. The cells exhibited a morphology characterized by a spindle-shaped form, compact cell bodies, and elongated processes (Figure 3).

### 3.4 Effects of catalpol on LPS-induced apoptosis in BV2 cells

**FIGURE 4:**
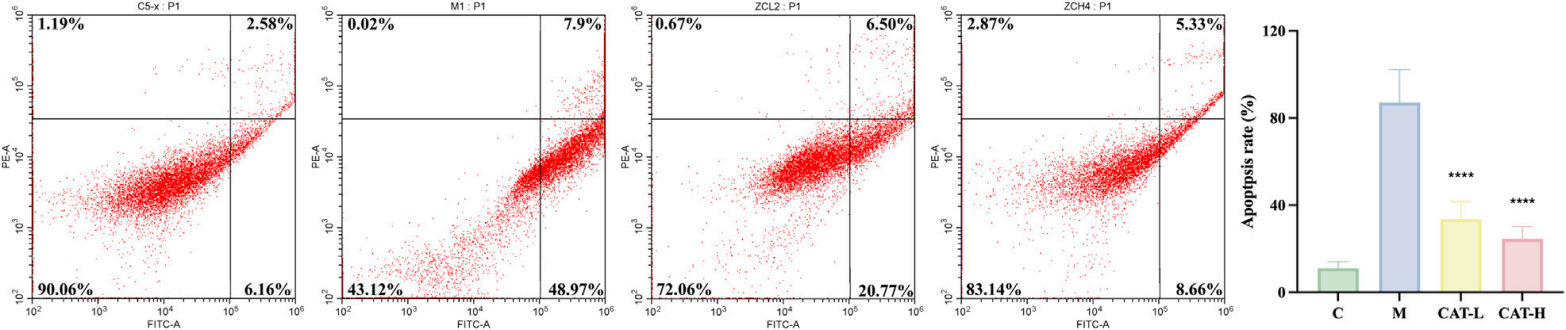
Catalpol abrogated the LPS-enhanced apoptosis in BV2 cells. The expression of apoptosis rate was determined using flow cytometry. Q1, cell debris or mechanically damaged cells; Q2, later apoptotic cells; Q3, early apoptotic cells; Q4, live cells. All data are presented as the mean ± SD (*n* = 3). ****p < 0.0001.

The apoptotic cell percentages were assessed through flow cytometry. As depicted in Figure 4, stimulation with LPS substantially elevated the apoptotic rate in BV2 cells, an effect that was notably alleviated by prior treatment with catalpol. Catalpol pre-treatment nearly entirely counteracted the LPS-induced increase in apoptosis among BV2 cells.

### 3.5 Effects of NF-κB/NLRP3 on Catalpol’s impact on LPS-Induced BV2 cell activation

**FIGURE 5:**
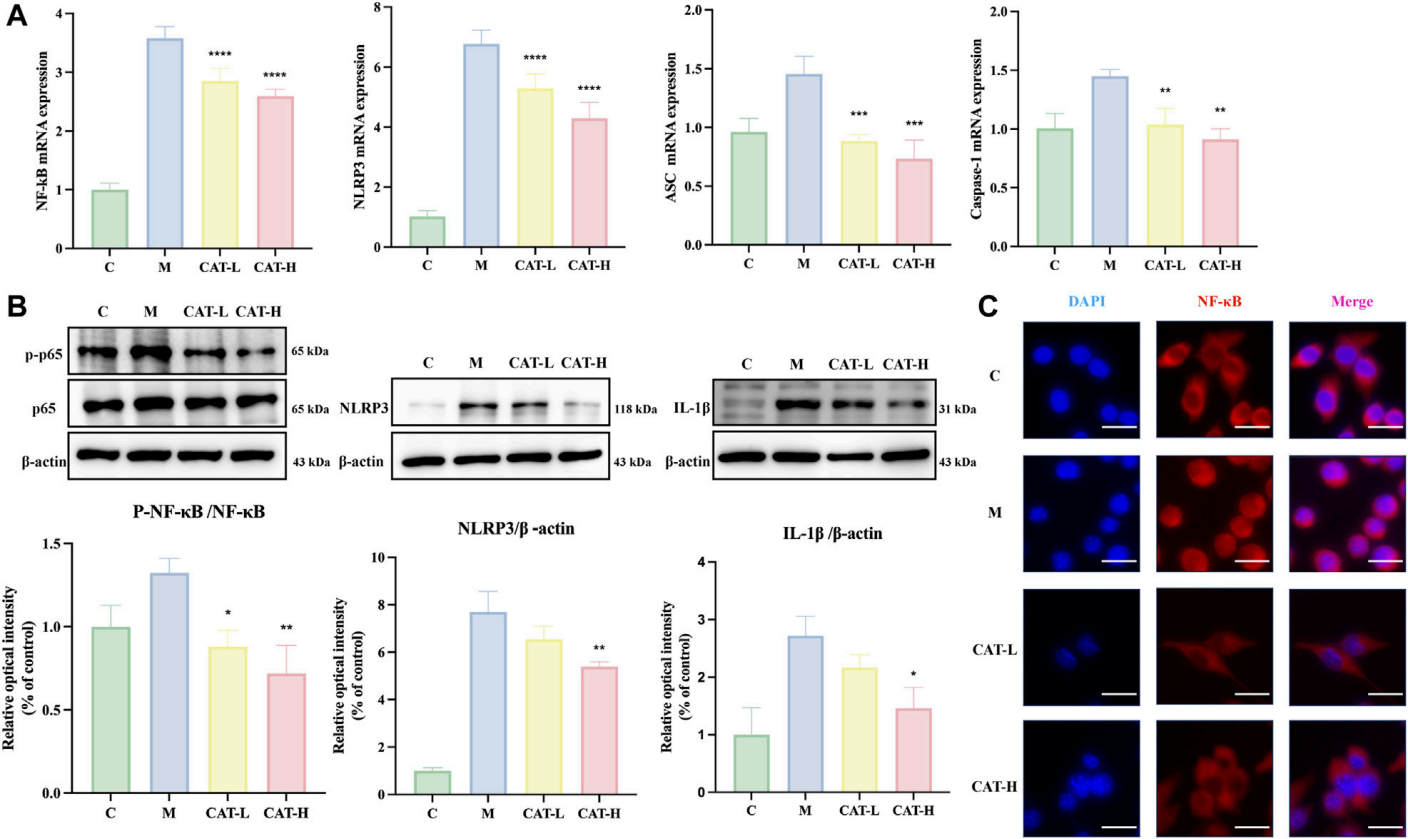
Catalpol effects on NF-κB/NLRP3 signaling in LPS-Induced BV2 cells. The whole cells were collected for assessing the gene expression of NLRP3, NF-κB, Caspase-1, and ASC using rt-qPCR **(A)**, and cells were collected to evaluate the protein expression of NF-κB p-65, p-NF-κB p-65, and NLRP3 via western blot analysis **(B)** Nuclear translocation of NF-κB p65 is significantly inhibited by catalpol in LPS-stimulated cells. Representative images of IF staining at ×20 magnification, Scale bar: 50 μM. **(C)** All data are presented as the mean ± SD (*n* = 3). *p < 0.05, **p < 0.01, ***p < 0.001 ****p < 0.0001.

To investigate whether catalpol influences microglial activation via the NF-κB/NLRP3 signaling pathway, P65, Phospho-P65, NLRP3, and IL-1β (downstream genes of the NF-κB pathway) were selected as targets for further experiments. Phospho-P65 was considered to be an indicator of NF-κB activation. In BV2 microglia, the mRNA levels of NLRP3, NF-κB, Caspase1, and ASC exhibited a significant increase in response to LPS treatment. However, Catalpol administration markedly reduced the expression levels of these genes (Figure 5A). Additionally, the protein levels were evaluated using western blots. As illustrated in Figure 5B, the LPS group exhibited a substantial increase in the expression levels of phosphorylated P65, NLRP3, and IL-1β, whereas catalpol pretreatment remarkably suppressed these effects. In addition, an immunofluorescence assay revealed that catalpol significantly reduced the level of P65 in the nucleus of BV2 induced by LPS (Figure 5C).

### 3.6 Molecular docking

**FIGURE 6:**
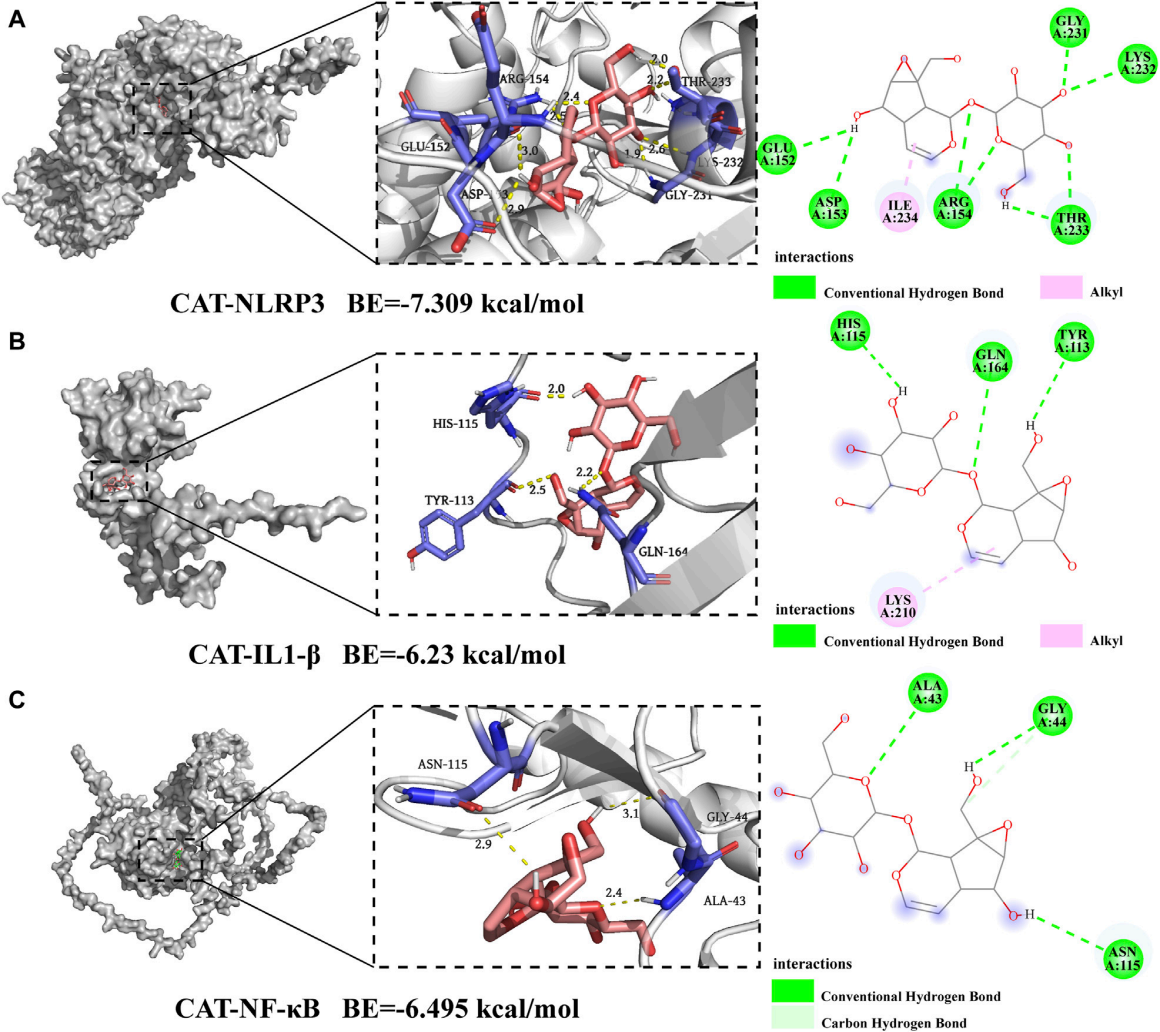
Molecular docking of catalpol with NLRP3, IL-1β, and NF-κB. **(A)** 2D and 3D binding models of NLRP3 and catalpol. **(B)** 2D and 3D binding models of IL-1β and catalpol. **(C)** 2D and 3D binding models of NF-κB and catalpol.

The more robust the interaction between the ligand and the receptor, the more negative the binding energy of the two. Three candidate target proteins, including NLRP3, IL-1β, and NF-κB, were conducted molecular docking with catalpol; the corresponding 2D-chemical structures were drawn using Discovery Studio software, whose results were visualized by Pymol. Our findings indicated that catalpol exhibited binding energies and the three core proteins were all ≤ −6 kcal/mol, the lowest binding free energy was NLRP3-catalpol (−7.309 kcal/mol), and the main forces involved were Alkyl, hydrophobic forces, and carbon-hydrogen bonds. Figure 6 shows the binding mode of catalpol to each target. In detail, for NLRP3 (Figure 6A), the compound was bound to the activity sites alkyl bonds with ILEA234. Moreover, catalpol could form hydrogen bonds with GLUA152, ASPA153, ARGA154, GLYA231, LYSA232, and THRA233. What’s more, more H-bonds than the other three proteins may explain the reason that NLRP3 showed the best binding energy. In the binding mode (Figure 6B), IL-1β was bound to the active binding sites via three hydrogen bonds and one alkyl interaction. In detail, the compound formed alkyl interactions with LYSA210. Moreover, Catalpol was bound to HISA115, GLNA164, and TYRA113 by three hydrogen bonds. For NF-κB as shown in Figure 6C, catalpol could form hydrogen bonds with ALAA43, GLYA44, and ASNA115 respectively. Together, catalpol could bind well with three core targets, all of which might play key roles in the influence of microglial activation.

### 3.7 Molecular dynamics simulations of catalpol

Given the inherent motion of proteins and small ligands, the conformation considered during molecular docking represents a relatively stable state for them. Molecular dynamics simulation is an extensively employed method for assessing the structural features of protein-ligand systems and examining the stability of binding between proteins and molecules. In this investigation, NF-κB, NLRP3, and IL-1β considered the most important targets in Catalpol against neuroinflammation, were selected for further assessment of the binding stability with Catalpol.

(1) RMSD analyses

**FIGURE 7:**
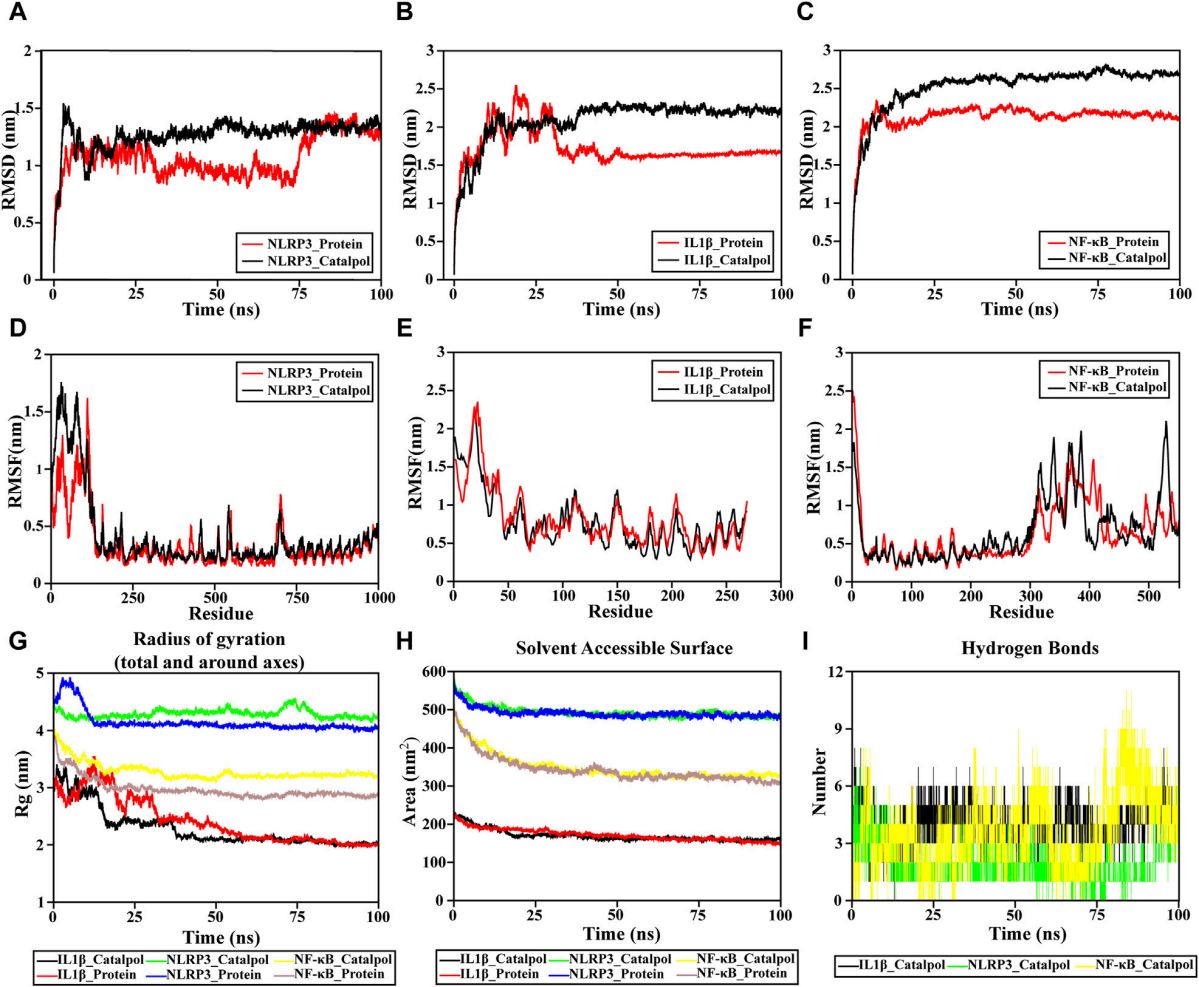
Molecular dynamic simulation. **(A–C)** RMSD of the complex MDS. **(D–F)** RMSF of the complex MDS is displayed in the high fluctuation range of the surface. **(G)** Rg variation diagram of the complex MDS. **(H)** SASA of the complex MDS. **(I)** HBNUM in the complex MDS.

The RMSD value of an individual protein and a protein-ligand complex can be compared to assess alterations in protein molecular dynamics and the conformational stability of the complex. The structural stabilization of the complex is indicated by a lower RMSD value of the protein-ligand complex compared to the solitary protein. In this study, the NLRP3-catalpol system had a sharp rise within 70 ns and tended to balance out the last 10 ns with the average RMSD value of 1.0748 ± 0.175. Meanwhile, the NLRP3 system was in an equilibrium state at 20–100 ns with a larger average RMSD value (1.2691 ± 0.1321), indicating that the stability of the catalpol-NLRP3 system was stronger (Figure 7A).

The IL-1β-catalpol complex was mostly stable throughout the simulation period. It attained a state of relative equilibrium at approximately 40 ns and remained stable until the end, with an average RMSD value of 2.0863 ± 0.286. The IL-1β system reached equilibrium after 50 ns, maintaining a smaller average RMSD value (1.7258 ± 0.2414), which reflects to a certain extent, the overall system stability. The equilibrium of both systems was achieved after a 50 ns simulation, indicating their satisfactory stability (Figure 7B).

The NF-κB-catalpol complexes experienced similar RMSD trends (Figure 7C). The NF-κB-catalpol complex rose to 2.3 nm at around 7 ns and remained stable till the end of NF-κB the simulation time with an average RMSD value of 2.132 ± 0.1665. The RMSD of NF-κB rose to 2.6 nm at 30 ns sustained stability until the conclusion of the simulation, with an average RMSD value of 2.5358 ± 0.3015 nm.

The absence of a break in the RMSD curve indicates that the compounds can firmly attach to proteins without dissociating from the protein pocket during the simulation. The RMSD outcomes for all complexes are noteworthy, indicating that small molecules can form stable bindings with proteins and sustain a relatively stable state.

(2) RMSF analyses

The RMSF plot serves as a typical representation of residues that underwent significant changes during the MD simulation process. Peaks in the RMSF diagram signify residues experiencing pronounced oscillations throughout the simulation. Additionally, elevated RMSF values suggest greater flexibility in protein domains. The binding of the drug to the protein typically induces a reduction in protein flexibility, thereby achieving stabilization of the protein and facilitating enzymatic activity.

The RMSF of the NF-κB and NLRP3 proteins upon binding with catalpol are consistently low, indicating the overall rigidity of the protein as demonstrated in Figure 7D–F. However, for IL-1β, the effect of catalpol on protein RMSF was not different. The overall RMSF results reveal that these complexes are sufficiently stable.

(3) Radius of gyration (Rg) analysis

The radius of gyration, Rg, serves as a crucial parameter for quantifying the structural variability of a protein during molecular dynamics (MD) simulations. Lower Rg values indicate a more rigid structure during the simulation. The Rg values (Figure 7G) decrease gradually in all systems, except NLRP3-catalpol, showing that the other two protein structures are compressed when binding to catalpol. The Rg of IL-1β-catalpol decreases from 3.4091 nm to 1.9695 nm with an average Rg value of 2.2852 ± 0.3315 nm. Similarly, the Rg of NFκB-catalpol is in the range of 4.02804 to 3.08936 nm, the average is 3.2839 ± 0.15nm, reflecting a reduced level of fluctuation in the system. The NLRP3-catalpol complex ranges from 4.5612 to 4.1304 nm, with a mean of 4.2908 ± 0.0729 nm, and in the last 20 ns, the Rg of the protein is stable at around 4.200 nm. The results of Rg show that these systems are compact and converged well.

(4) Protein Solvent Accessible Surface Area (SASA)

Protein Solvent Accessible Surface Area (SASA) is considered a crucial element in investigations related to protein folding and stability. A decreased SASA value signifies enhanced compactness.

The solvent-accessible surface area of NLRP3-catalpol, IL-1β-catalpol, and NF-κB-catalpol complexes gradually decreased throughout the simulation, indicating that the bindings between them are gradually increased (Figure 7H).

(5) H-bond numbers analyses

Hydrogen bonds represent one of the most robust non-covalent binding interactions. The number of hydrogen bonds in the protein-compound complexes reflected their binding strengths. The larger the number, the better the binding.

The findings indicate a notably higher count of hydrogen bonds between catalpol and NF-κB compared to those formed with NLRP3. Among them, NF-κB-catalpol had the highest hydrogen bond density and strength, followed by IL-1β-catalpol, and NLRP3-catalpol (Figure 7I).

### 3.8 Binding free energy analysis

The Molecular Mechanics Poisson–Boltzmann Surface Area (MM/PBSA) method serves as a robust and dependable approach for computing the binding free energy of small molecule compounds to their respective protein targets. Typically, complexes exhibiting lower binding free energy are deemed more stable, suggesting that their ligands are likely to possess increased activity and potency.

**FIGURE 8:**
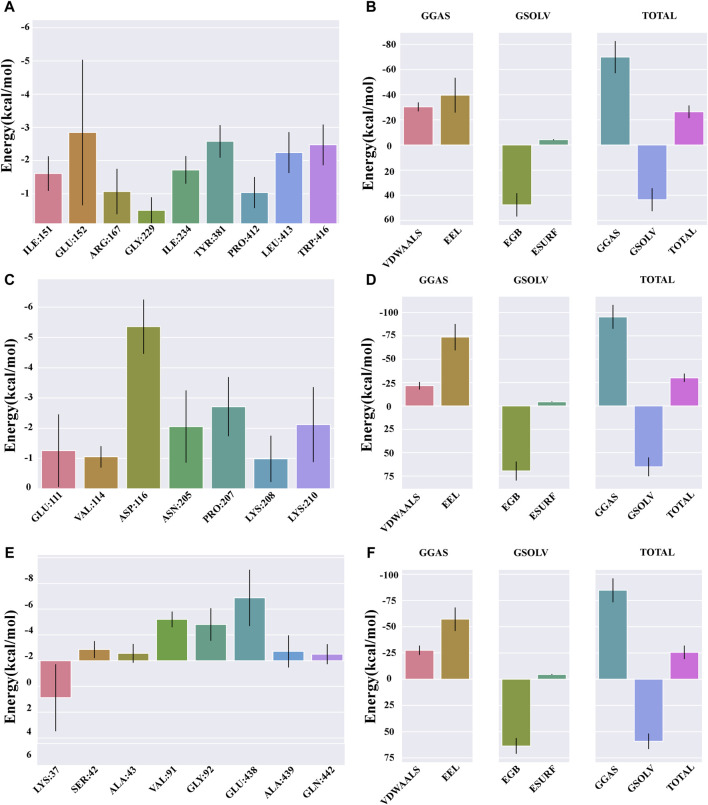
Binding free energy analysis. **(A)** Energy amino acid decomposition diagram for NLRP3–catalpol. **(B)** Energy attribute breakdown diagram for NLRP3–catalpol. **(C)** Energy amino acid decomposition diagram for IL-1β–catalpol. **(D)** Energy attribute breakdown diagram for IL-1β-catalpol. **(E)** Energy amino acid decomposition diagram for NF-κB–catalpol. **(F)** Energy attribute breakdown diagram for NF-κB–catalpol.

The protein-ligand affinity in the NLRP3 system was recorded as −26.4 kcal/mol (Figure 8B), whereas in the IL-1β system, it amounted to −30.14 kcal/mol (Figure 8D), and in the NF-κB system, it was −25.51 kcal/mol (Figure 8F).

The free energy residue decomposition diagram provides insights into the amino acid residues that contribute to the binding during ligand simulation. The key residues contributing towards the binding interaction between NLRP3 and catalpol include ILE151 (−1.61 kcal/mol), GLU152 (−2.85 kcal/mol), ARG167 (−1.07 kcal/mol), ILE234 (−1.72 kcal/mol), TYR381 (−2.58 kcal/mol), PRO412 (−1.04 kcal/mol), LEU413 (−2.24 kcal/mol), and TRP416 (−2.47 kcal/mol) (Figure 8A). In case of IL-1β-Catalpol complex, residues such as GLU111 (−1.26 kcal/mol), VAL114 (−1.05 kcal/mol), ASP116 (−5.36 kcal/mol), ASN205 (−2.05 kcal/mol), PRO207 (−2.71 kcal/mol), LYS210 (−2.12 kcal/mol) contribute majorly to the total binding energy (Figure 8C). In case of NF-κB-Catalpol complex, residues such as LYS37 (−2.88 kcal/mol), VAL91 (−3.2 kcal/mol), GLY92 (−2.81 kcal/mol), and GLU438 (−4.88 kcal/mol) have higher contribution toward the total binding energy (Figure 8E).

## 4 Discussion

Stroke stands as the second leading cause of mortality globally, accounting for approximately 12% of total deaths and imposing an escalating burden, especially in low-income countries (Feigin et al., 2021). Traditional Chinese Medicine (TCM) has a rich history of employing various medicinal herbs for stroke treatment, exhibiting robust therapeutic effects in both pharmacological experiments and clinical applications (Zhu et al., 2020). The responsive nature of microglia to injury suggests their potential as diagnostic markers for disease onset and progression, particularly given their involvement in neuroinflammation. Genetic studies have provided strong support for the pivotal role of microglia-mediated neuroinflammation in neurodegeneration (Perry et al., 2010; Crotti and Ransohoff, 2016; Rangaraju et al., 2018). Consequently, it is of paramount importance to identify bioactive ingredients in TCM that can regulate microglial function in the brain, thereby mitigating neurotoxic inflammatory mediators.

Prior research has underscored the anti-inflammatory characteristics of catalpol (Zhang H. et al., 2019; Yang et al., 2020; Wu et al., 2022; Ni et al., 2023). Additionally, researches have extensively explored catalpol for its potential effects in experimental models of various neurological disorders such as depression, Alzheimer’s, and Parkinson’s disease (Bhattamisra et al., 2019). In a specific study, patients experiencing depression exhibited heightened levels of pro-inflammatory cytokines, including interleukin-1β (IL-1β) and tumor necrosis factor-α (TNF-α). The inhibition of COX-2 led to a subsequent decrease in these heightened cytokine levels, and catalpol exhibited a notable suppressive effect on the levels of COX-2 and prostaglandin E2 (PGE2) in the frontal cortex and hippocampus of mice (Kuwano et al., 2004; Wang et al., 2015).

In our study, we employed LPS as an inflammation stimulant to investigate the effects of catalpol on LPS-induced neuroinflammatory responses. Our findings illustrated that catalpol treatment led to a notable inhibition of NO production in LPS-activated cells. This suggests that catalpol has the potential to mitigate inflammation by reducing excessive NO levels. Additionally, the triggering of proinflammatory cytokines (TNF-α, IL-1β, and IL-6) and the upregulation of Iba-1 upon LPS treatment contributed to brain neuroinflammation. Intriguingly, catalpol treatment significantly mitigated these LPS-induced neuroinflammatory changes, highlighting its potential as an anti-inflammatory agent.

We obtained a significant result in our study. Primarily, catalpol significantly suppressed LPS-induced neuroinflammation by reducing the production of pro-inflammatory cytokines, phosphorylation of NF-κB, expression of Iba-1, and regulating NLRP3 to inhibit inflammasome activation. Catalpol indeed has broad medicinal value and application prospects due to its effects on multiple organs and tissues. However, most research on catalpol remains at the basic experimental stage, with clinical evaluations primarily focused on its anti-tumor activity (Fei et al., 2018). Other pharmacological effects have yet to be thoroughly evaluated in clinical settings. Therefore, more research is needed to establish a solid foundation for clinical trials and expedite its clinical application.

To clarify the underlying mechanisms involved in inhibiting the release of inflammatory factors and modulating microglial phenotypes, we investigated the activation of NF-κB/NLRP3 signaling pathways induced by LPS. LPS, a component found in the outer membrane of Gram-negative bacteria, functions as a ligand for TLR4, triggering several downstream signal pathways, including NF-κB (Nair et al., 2019). The NF-κB transcription factor is prominently expressed in various brain cells, encompassing neurons, microglia, and astrocytes. It’s vital role lies in the regulation of inflammatory genes and its involvement in a multitude of brain functions (Sun et al., 2022). NF-κB activation entails the movement of the P50/P65 subunits of NF-κB from the cytoplasm to the nucleus in the majority of cell types (Tak and Firestein, 2001). Numerous research investigations have suggested that inhibiting NF-κB could serve as a pivotal factor in halting the progression of ischemic stroke (IS) pathology.

The NLRP3 inflammasome, consisting of multiple proteins, is recognized for its ability to activate procaspase-1. This activation subsequently triggers the cleavage and secretion of IL-1β and IL-18 (Lamkanfi and Dixit, 2014). Activation of NLRP3 involves two signals. The first is initiated by the binding of the TLR4 receptor to its ligand LPS, subsequently triggering NF-κB-mediated upregulation of NLRP3 along with proIL-1β (Bauernfeind et al., 2009). Upon activation, NLRP3 assembles into an inflammasome complex with the adaptor molecule ASC, regulating the activation of Caspase-1 (Dinarello, 2009). Extensive research has demonstrated the association between NLRP3 inflammasome activation and neuroinflammation in neurodegenerative disorders. Recent evidence has also proposed a potential connection between the increased activity of the inflammasome and neuronal/glial cell death in cerebral ischemia (Fann et al., 2013). Additionally, a recent study has proposed that NLRP3 deficiency distinctly alleviated microglial activation in mice (Tejera et al., 2019; Wu et al., 2021). Furthermore, there is another mechanism of inflammasome activation known as the non-canonical inflammasome pathway, triggered by the cytosolic sensing of LPS (Diamond et al., 2015). The non-canonical inflammasome also serves as a signal for activating the canonical inflammasome, playing a crucial role in inducing and promoting inflammatory responses (Chi et al., 2014; Down et al., 2020). A recent study has shown the activation of a novel NLRP1-ASC-caspase-8 non-canonical inflammasome in the mouse cortex during inflammation (Cyr et al., 2022). However, research on the non-canonical pathway in the context of ischemic stroke is relatively scarce, which is a limitation of our study. Future research should focus on understanding the mechanisms of the non-canonical inflammasome pathway in ischemic stroke, as this will be crucial for advancing our knowledge in this area.

The NF-κB/NLRP3 signaling pathways exert crucial functions in controlling the expression of proinflammatory cytokines in microglia following LPS stimulation (Lam et al., 2001). Consequently, we explored whether the suppressive impact of catalpol on the NF-κB/NLRP3 pathway could provide neuroprotective benefits against LPS-induced neuroinflammatory responses in BV2 cells. As anticipated, the outcomes showed a pronounced increase in the phosphorylation of NF-κB P65 in BV2 cells following LPS stimulation. However, catalpol administration successfully mitigated the hyperactivity of NF-κB induced by LPS in BV2 cells. Secondly, our data demonstrate a notable reduction in LPS-induced NF-κB P65 nuclear translocation upon catalpol administration in microglial cells. Therefore, catalpol may mitigate the LPS-induced neuroinflammatory response by restoring the balance of NF-κB activation and inhibiting NF-κB P65 nuclear translocation. The NF-kB signaling pathway plays a crucial role in triggering the activation of NLRP3 (Bauernfeind et al., 2011; Lamkanfi and Dixit, 2014). Furthermore, our results indicate that catalpol attenuated LPS-induced neuroinflammation by suppressing NLRP3 inflammasome signaling. More specifically, catalpol administration resulted in the inhibition of NLRP3 activation, caspase-1 cleavage, and ASC expression. Mechanistically, our results demonstrated that catalpol could alleviate the inflammatory response in microglia by downregulating the NF-κB and NLRP3 inflammasome pathways.

Progress in science and technology has facilitated the application of computational analyses and simulations in research, thereby surmounting experimental constraints and augmenting research efficiency This pivotal role has been instrumental in consolidating and propelling advancement in neuroinflammation research (Li et al., 2021).

This study involved molecular docking of catalpol with NLRP3, IL-1β, and NF-κB to investigate its pharmacological targets. Notably, catalpol, known for its efficacy against neuroinflammation, exhibited robust binding affinity to these three targets, highlighting its significant role in inflammation regulation. Additionally, we utilized MD simulation, a potent tool for assessing the stability of protein-ligand complexes and visualizing structural dynamics during the simulation period. This approach was instrumental in evaluating the stability of catalpol binding to NLRP3, IL-1β, and NF-κB in their respective binding pockets over a 100 ns duration.

Lower RMSD values indicate greater structural stability. In the case of the catalpol-NLRP3 system, a lower RMSD suggests robust stability. An illustrative representation of residue changes during MD simulation is the RMSF plot. Our RMSF results showed that NF-κB and NLRP3, when bound to small molecules, experienced generally low overall RMSF, indicating increased rigidity upon binding to small molecules and enhancing the overall protein structure stability. The radius of gyration (Rg) is another parameter used to measure structural fluctuations during MD simulations. For NF-κB-catalpol and IL-1β-catalpol, Rg suggested stable fluctuations in size, implying high binding potential. SASA is considered a crucial element in researching protein folding and stability. Hydrogen bond analysis, reflecting binding strengths, showed that IL-1β-catalpol and NF-κB-catalpol exhibited superior hydrogen bonding in terms of size and density. All three complexes demonstrated the ability to form stable hydrogen bonds, establishing a solid foundation for stable bonding.

Free energy analysis showed similar sign distribution for NLRP3, IL-1β, and NF-κB. However, the IL-1β affinity was stronger at −30.14 kcal/mol compared to NLRP3 and NF-κB. Stable binding was revealed through MDS, supporting the anti-neuroinflammatory role of catalpol via multiple targets. Furthermore, the breakdown diagram of free energy residues can be utilized to comprehend which amino acid residues contribute to the binding during ligand simulation. These findings, in turn, provide additional support to the earlier experiments.

Emerging evidence strongly implicates neuroinflammation as a pivotal mechanism in neurodegenerative diseases, including ischemic stroke (Stephenson et al., 2018; Endres et al., 2022; Amanollahi et al., 2023). The overactivation of microglia leads to the secretion of proinflammatory mediators, resulting in neuroinflammation. Moreover, augmented neuroinflammatory responses mediated by glial cells and elevated levels of pro-inflammatory mediators have been observed in both animal models of ischemic stroke and IS patients (Price et al., 2006; Ormstad et al., 2011; Ding et al., 2022; Cui et al., 2023). Therefore, targeting the regulation of microglial cell-mediated neuroinflammation represents a promising therapeutic approach for neurodegenerative diseases. In this study, we demonstrated that catalpol effectively modulates LPS-induced neuroinflammation in BV-2 cells. Collectively, our results highlight that catalpol can effectively regulate glia-induced neuroinflammatory responses, providing potential anti-inflammatory effects in the brain. Consequently, it may offer an effective treatment for neuroinflammation-related diseases, including neurodegenerative diseases such as ischemic stroke.

Although this study demonstrates the beneficial effect of catalpol on neuroinflammation and its regulatory role on NF-κB/NLRP3, given the intricate pathology and progression of IS injury, our study represents only a preliminary investigation. A thorough exploration of the potential mechanisms is needed in future research endeavors. Unfortunately, due to time constraints and other practical factors, we were unable to include a more extensive *in vivo* mechanism study in this paper. As a result, the *in vivo* experimental results cannot be presented in this manuscript. However, we are currently undertaking comprehensive *in vivo* studies to further investigate the neuroprotective effects of catalpol against ischemic stroke (IS).

To explore the anti-neuroinflammatory effects of catalpol in more detail, we are establishing a rat middle cerebral artery occlusion/reperfusion (MCAO/R) model. This will involve using TTC staining to calculate the volume of cerebral infarction, H&E staining and Nissl staining to observe histopathological changes, and immunofluorescence staining to assess the expression of IBA-1. Additionally, we will employ qRT-PCR and Western blots to measure the expression of inflammatory factors at both the gene and protein levels. These experiments aim to observe the protective effects of catalpol in the rat MCAO model and to further elucidate its underlying mechanisms. Future research endeavors will be crucial in providing deeper insights into the mechanisms by which catalpol exerts its neuroprotective effects in the context of IS.

## 5 Conclusion

**FIGURE 9:**
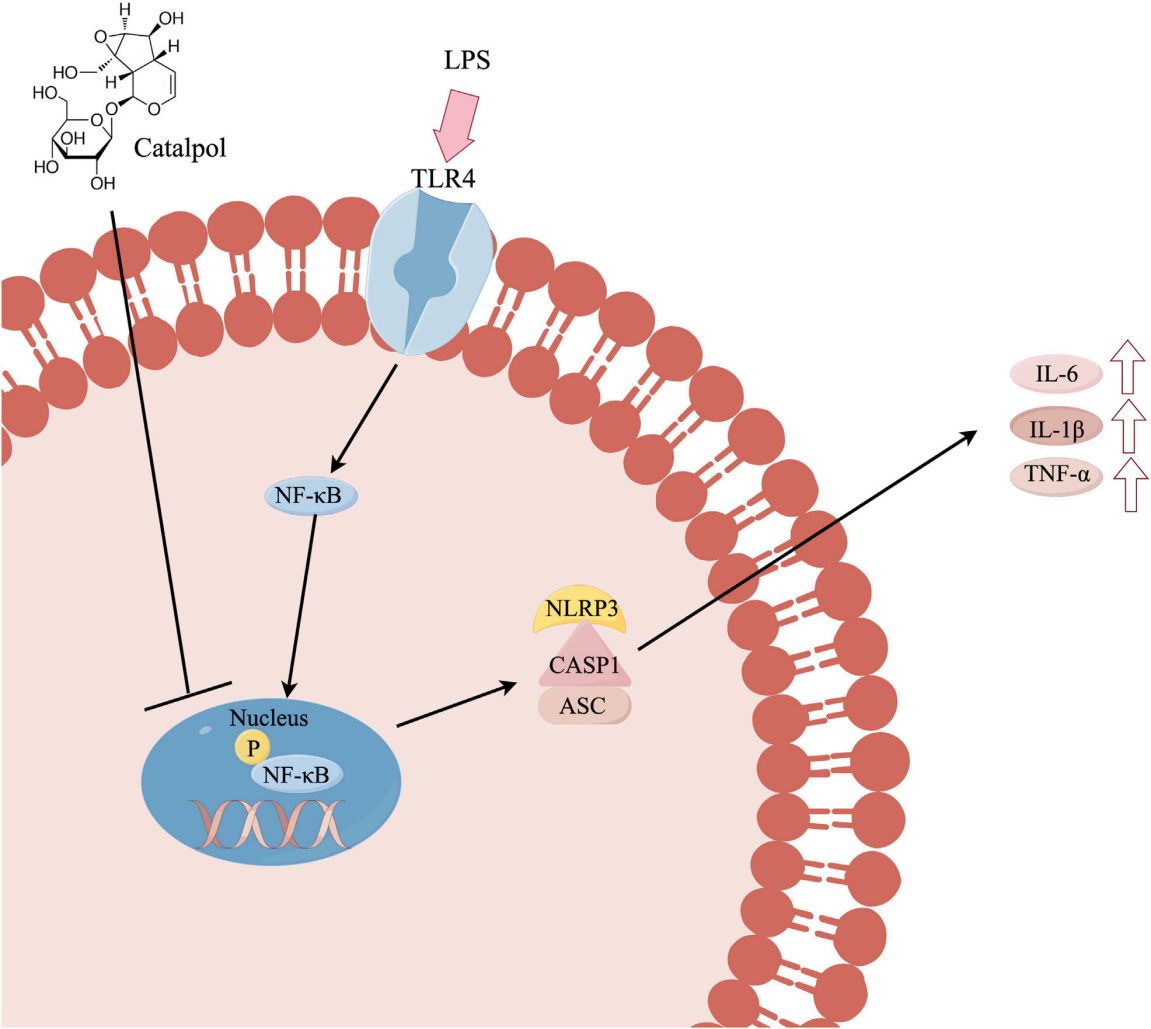
Schematic potential cellular mechanisms By Figdraw.

Catalpol exhibited anti-inflammatory activity by attenuating inflammatory markers in BV-2 microglia. This inhibitory effect may be attributed to the suppression of the NF-κB/NLRP3 pathway. Schematic potential cellular mechanisms as shown in Figure 9.

## Data Availability

The original contributions presented in the study are included in the article/Supplementary Material, further inquiries can be directed to the corresponding author.
