# Supplementary figures and images for: Catalpol reduced LPS induced BV2 immunoreactivity through NF-κB/NLRP3 pathways: an in Vitro and in silico study
Source: Front Pharmacol. 2024 Jun 27;15:1415445. doi: 10.3389/fphar.2024.1415445 (PMC11237369; doi:10.3389/fphar.2024.1415445)

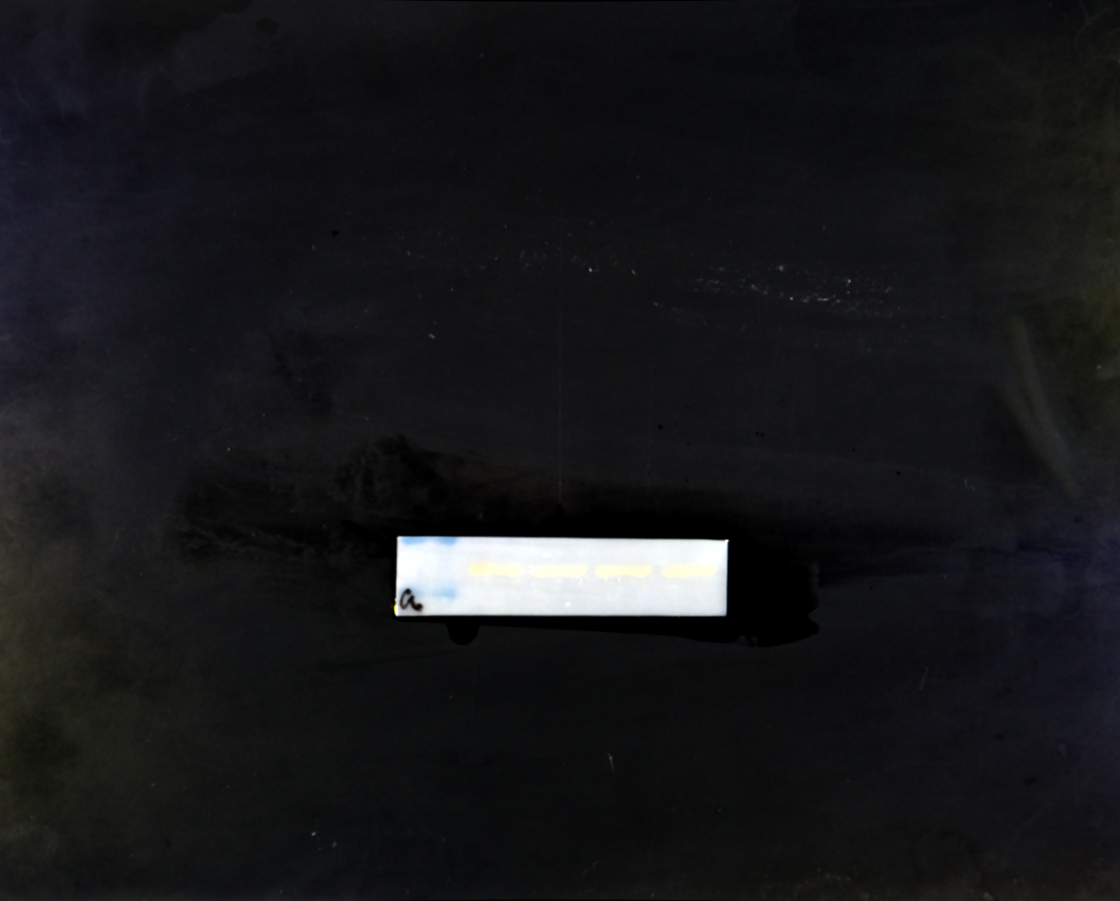

Supplement: Supplementary file 1 [file Image6.TIF]

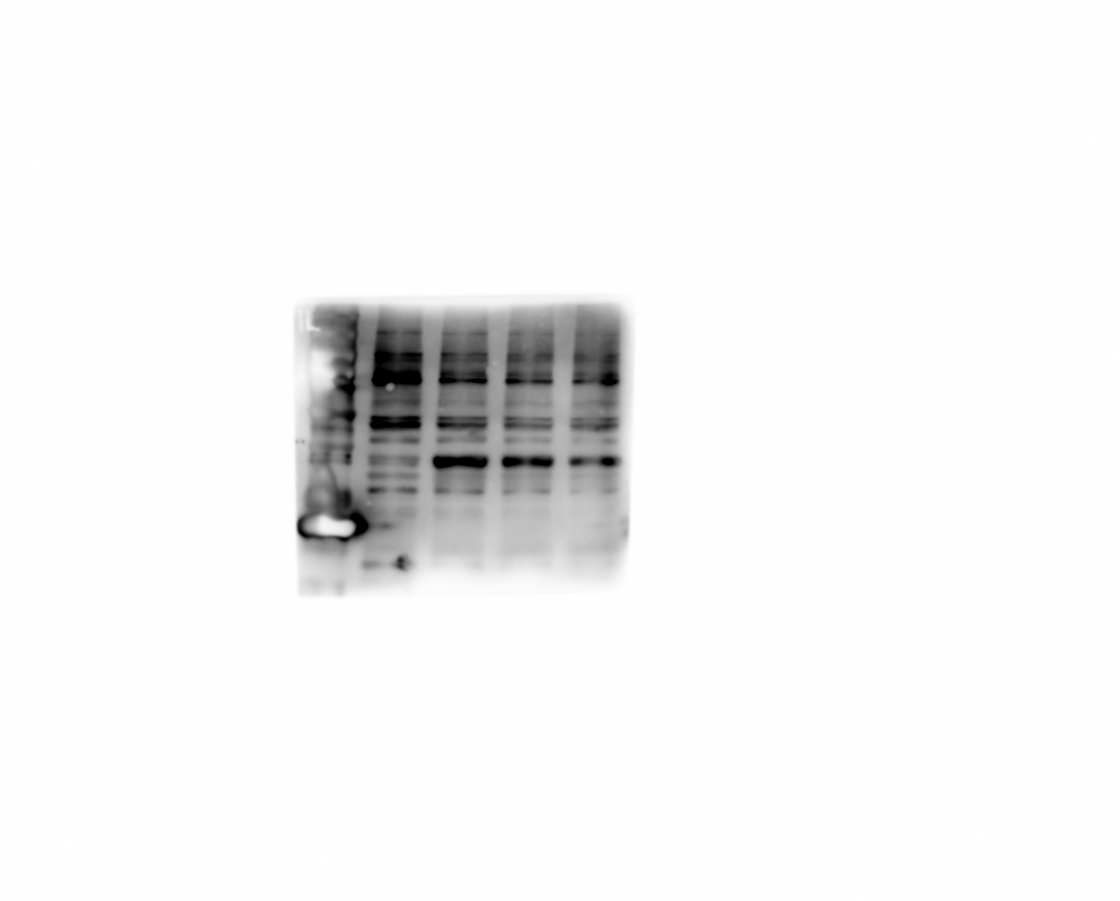

Supplement: Supplementary file 3 [file Image3.TIF]

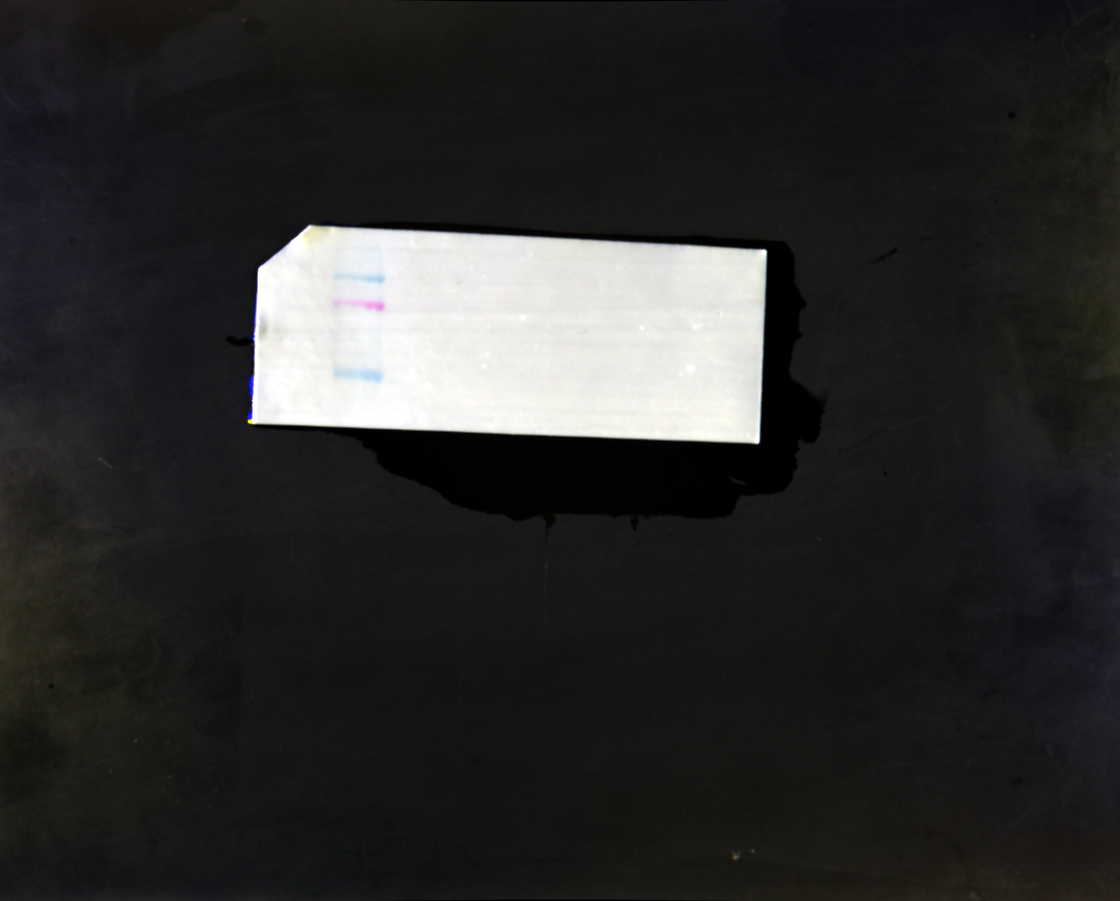

Supplement: Supplementary file 4 [file Image4.TIF]

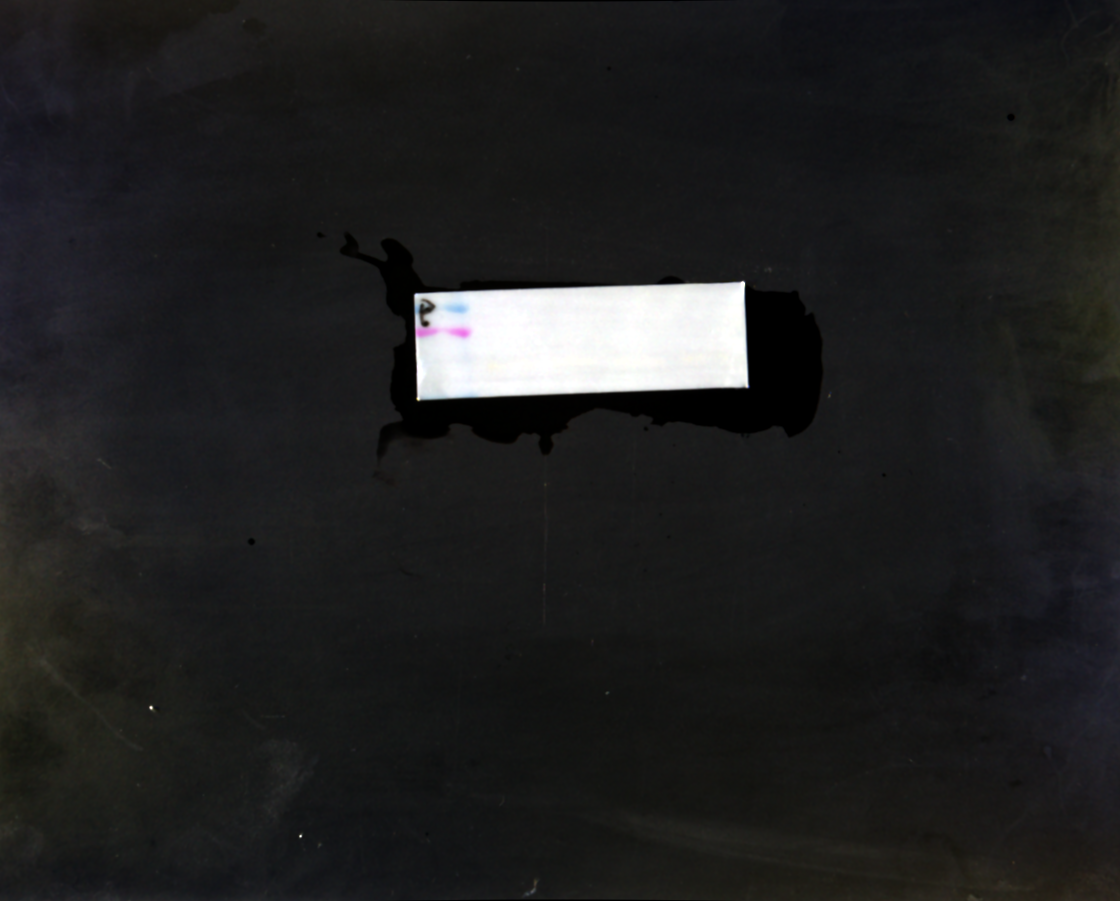

Supplement: Supplementary file 5 [file Image9.TIF]

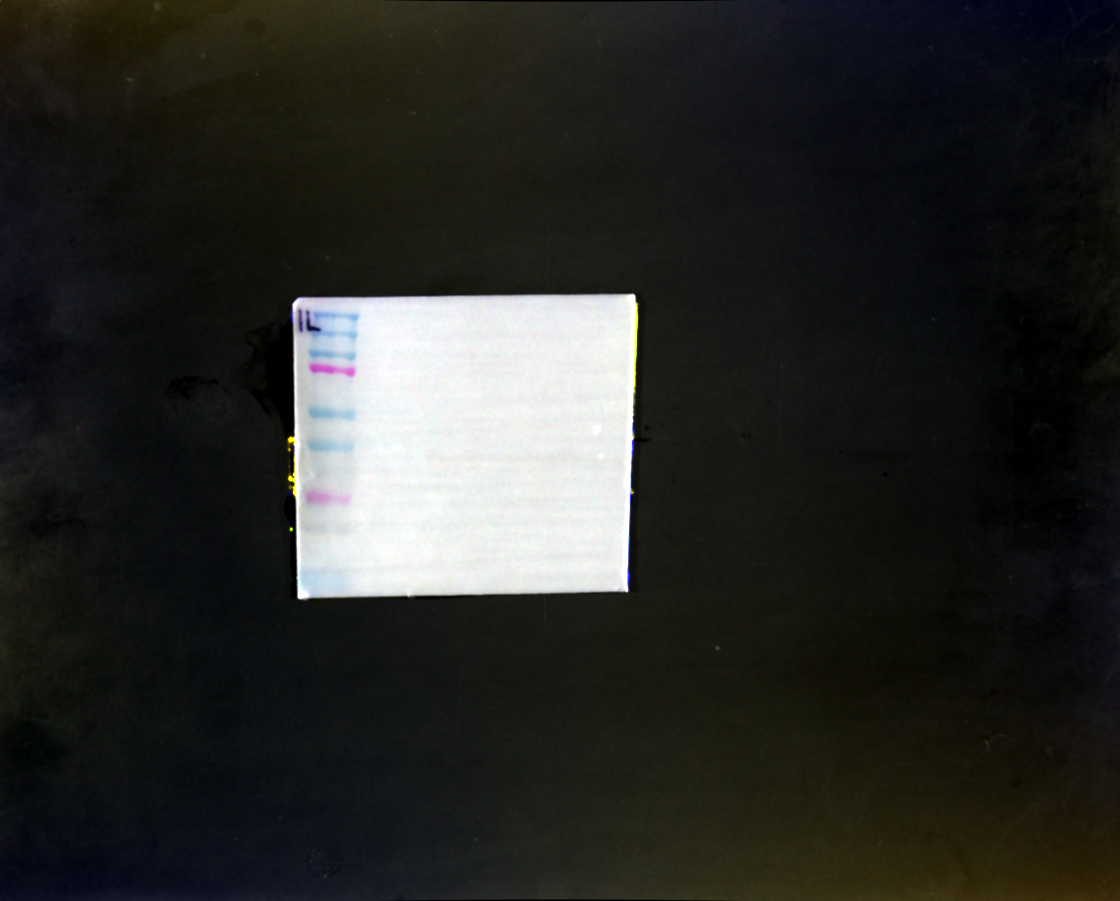

Supplement: Supplementary file 6 [file Image2.TIF]

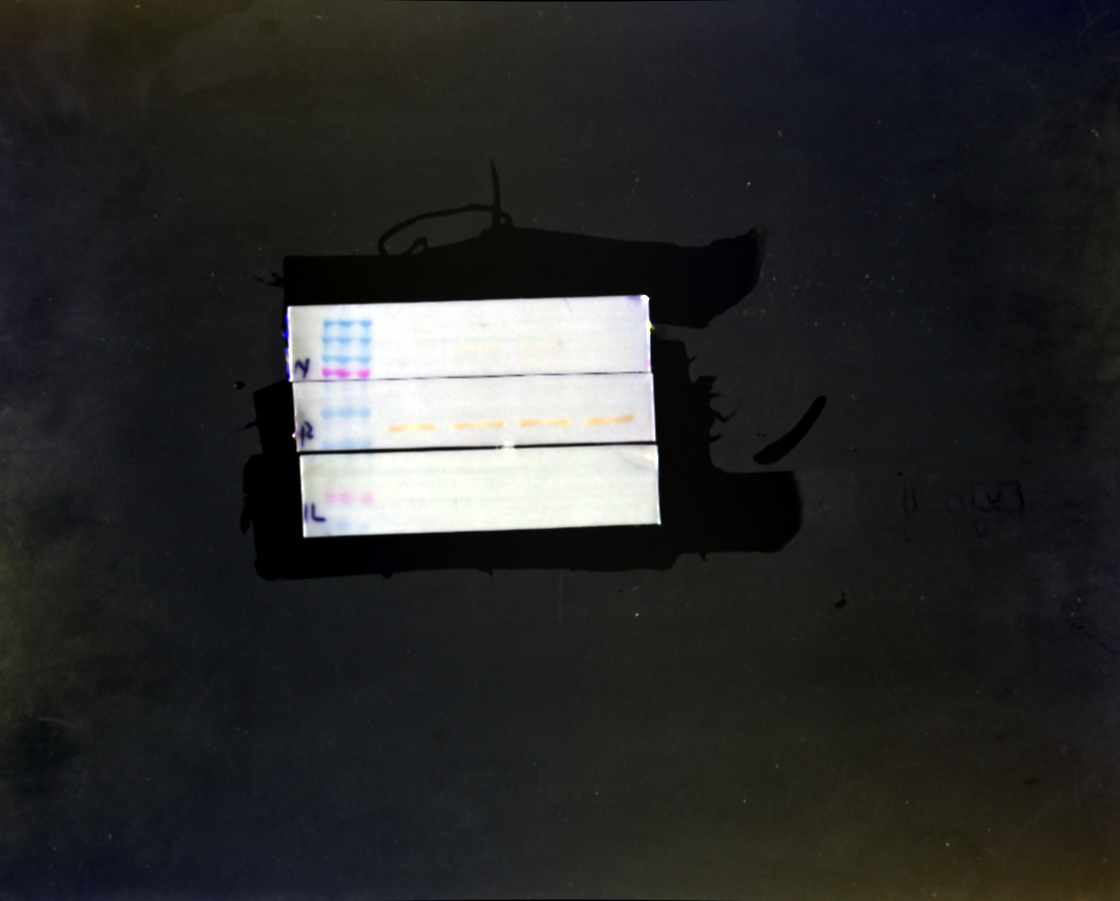

Supplement: Supplementary file 7 [file Image11.TIF]

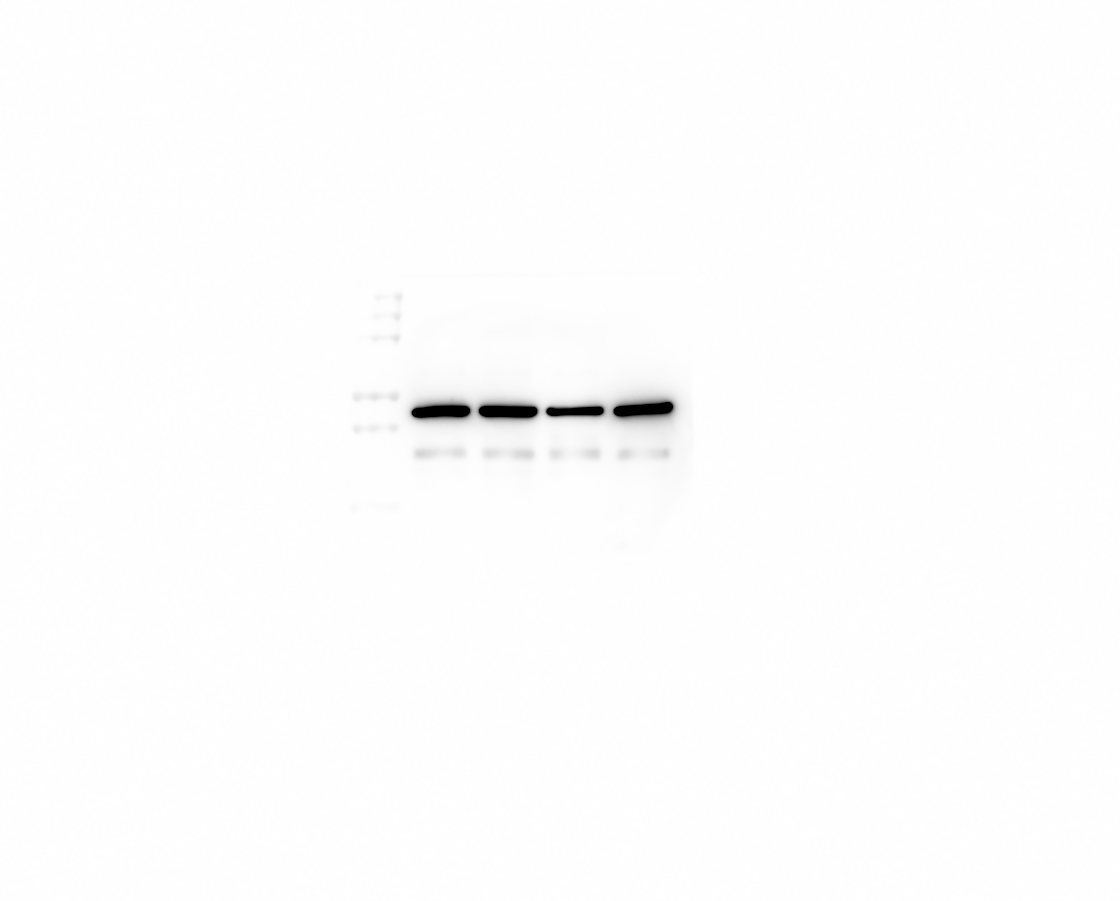

Supplement: Supplementary file 8 [file Image1.TIF]

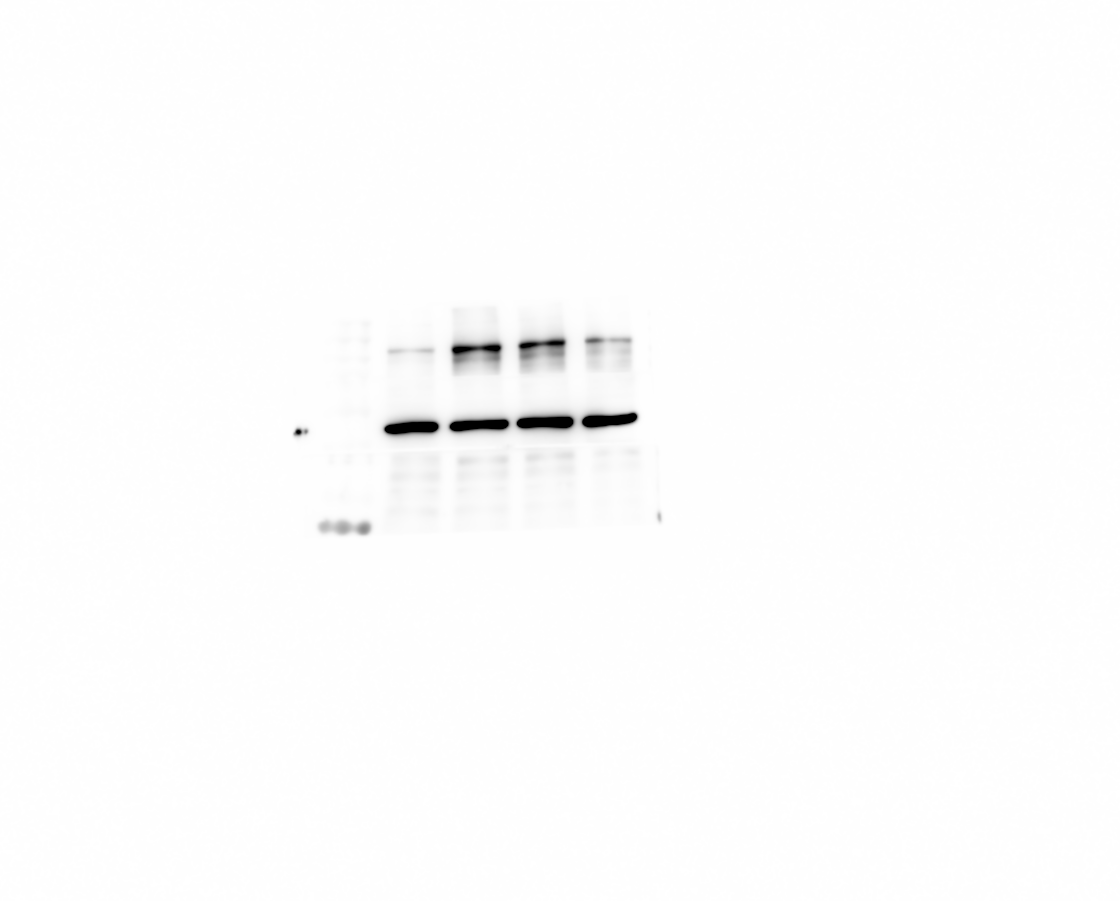

Supplement: Supplementary file 9 [file Image10.TIF]

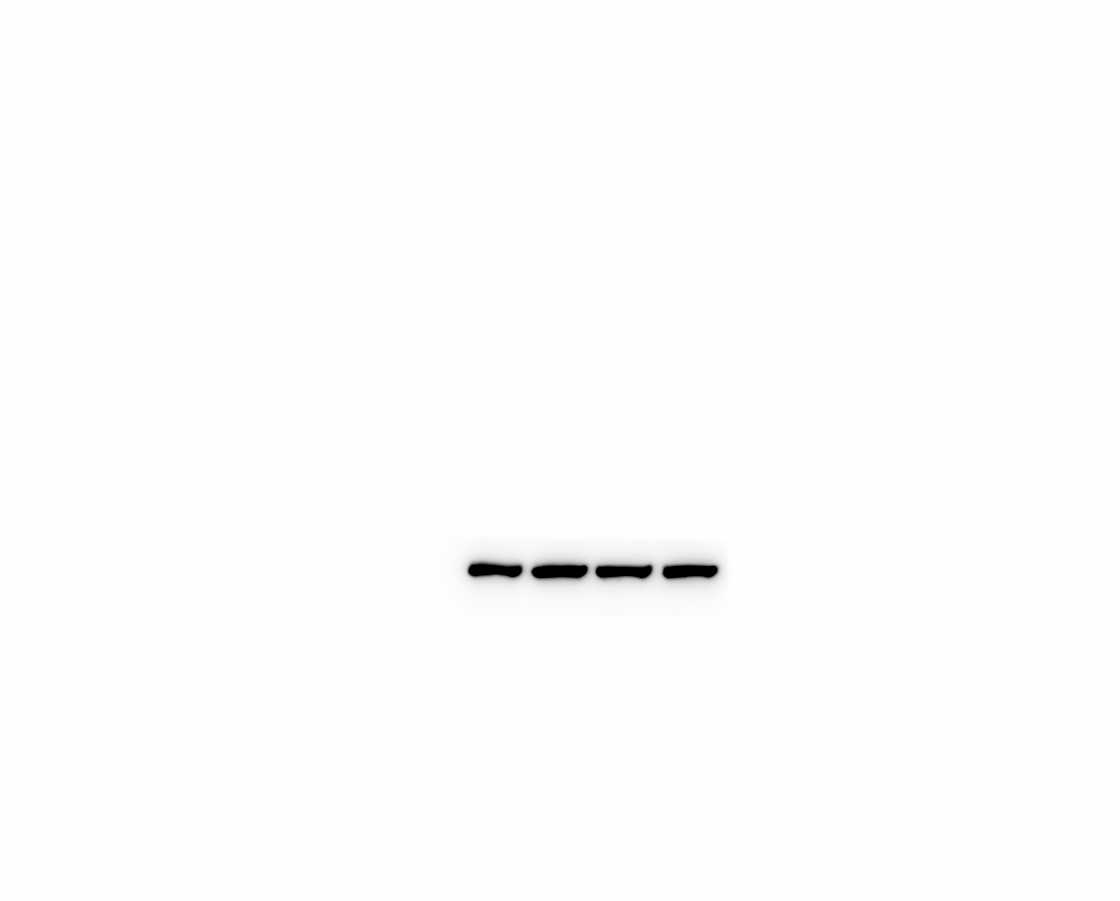

Supplement: Supplementary file 10 [file Image7.TIF]

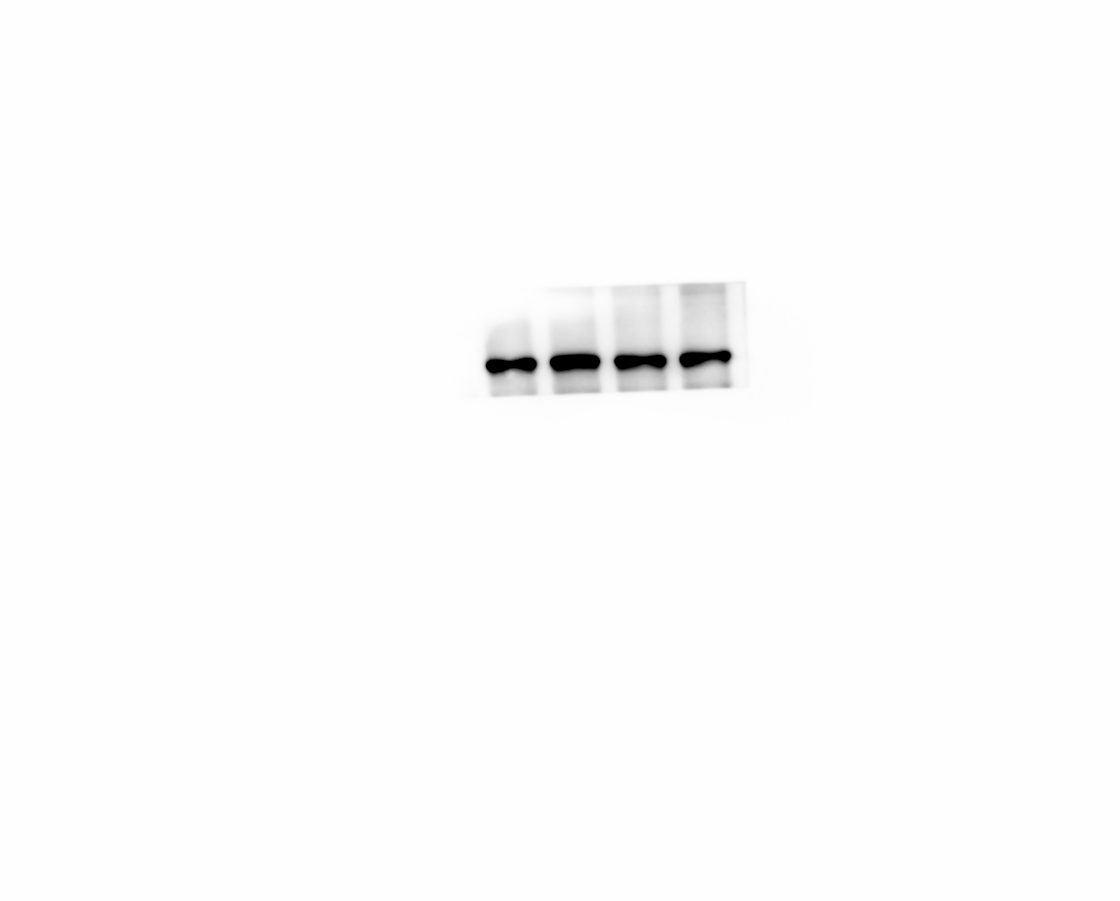

Supplement: Supplementary file 12 [file Image8.TIF]

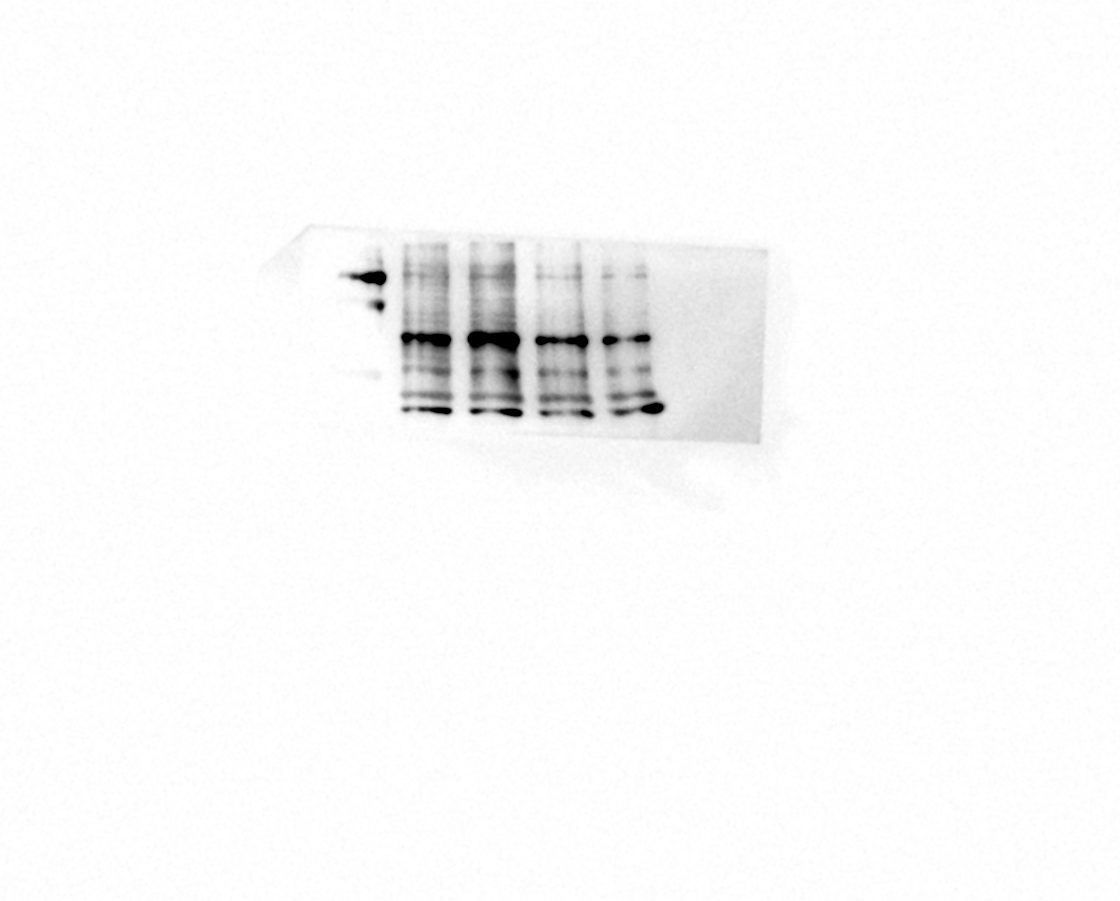

Supplement: Supplementary file 13 [file Image5.TIF]
