# Supplementary material for: Catalpol reduced LPS induced BV2 immunoreactivity through NF-κB/NLRP3 pathways: an in Vitro and in silico study
Source: Front Pharmacol. 2024 Jun 27;15:1415445. doi: 10.3389/fphar.2024.1415445 (PMC11237369; doi:10.3389/fphar.2024.1415445)
Supplement: Supplementary file 2 [file Table1.docx]

Supplementary Material

**Table 1** Sequences of the primers for qRT-PCR.

| **GENE** | **Primer sequences** |
| --- | --- |
| NF-κB | F: GCTGCCAAAGAAGGACACGACA  R: GGCAGGCTATTGCTCATCACAG |
| NLRP3 | F: TCACAACTCGCCCAAGGAGGAA  R: AAGAGACCACGGCAGAAGCTAG |
| ASC | F: CTGCTCAGAGTACAGCCAGAAC  R: CTGTCCTTCAGTCAGCACACTG |
| Caspase-1 | F: GGCACATTTCCAGGACTGACTG  R: GCAAGACGTGTACGAGTGGTTG |
| IL-1β | F: CACTACAGGCTCCGAGATGAACAAC  R: TGTCGTTGCTTGGTTCTCCTTGTAC |
| IL-6 | F: CTTCTTGGGACTGATGCTGGTGAC  R: TCTGTTGGGAGTGGTATCCTCTGTG |
| TNF-α | F: CTATGGCCCAGACCCTCACA  R: TCTTGACGGCAGAGAGGAGG |
| GAPDH | F: TGGGCTACACTGAGGACCACT  R: GGGAGTGTCTGTTGAAGTCG |
