# Supplementary material for: Catalpol reduced LPS induced BV2 immunoreactivity through NF-κB/NLRP3 pathways: an in Vitro and in silico study
Source: Front Pharmacol. 2024 Jun 27;15:1415445. doi: 10.3389/fphar.2024.1415445 (PMC11237369; doi:10.3389/fphar.2024.1415445)
Supplement: Supplementary file 11 [file Table2.docx]

Supplementary Material

**Table 2** The antibodies used in this study, including their names, catalog numbers, manufacturers, and dilution ratios:

| **Antibody Name** | | **Catalog Number and Company** | **Dilution Ratio** |
| --- | --- | --- | --- |
| NF-κB | 8242, CST | | 1:1000 |
| p-NF-κB | 3033, CST | | 1:1000 |
| NLRP3 | Ab270449, Abcam | | 1:1000 |
| IL-1β  Iba-1  β-actin | 16806-1-AP, Proteintech  SAB2702364, Merck  4979, CST | | 1:1000  1:1000  1:2000 |
